# Building Up a Biomedical Research Workforce Trial

**DOI:** 10.1017/cts.2025.10144

**Published:** 2025-09-10

**Authors:** Doris M. Rubio, Gretchen E. White, Audrey J. Murrell, Pearl Nielsen, Natalia E. Morone

**Affiliations:** 1 Department of General Internal Medicine, School of Medicine, University of Pittsburgh, Pittsburgh, PA, USA; 2 Joseph M. Katz Graduate School of Business and College of Business Administration, University of Pittsburgh, Pittsburgh, PA, USA; 3 Chobanian & Avedisian School of Medicine, Boston University, Boston, MA, USA

**Keywords:** Psychological Capital, career development, cluster randomized controlled trial, career advancement for underrepresented groups, academic medical centers

## Abstract

**Introduction:**

Scientific teams that are comprised of different types of researchers have higher research productivity, and there is a need for evidence-based methods to improve the biomedical research workforce. Building Up a Biomedical Research Workforce (Building Up) was a multi-center, cluster-randomized, unblinded controlled trial with one intervention arm and one control arm, conducted at 25 United States academic medical centers. The authors tested the hypothesis that participants from backgrounds underrepresented in science who are randomized to the intervention will have greater numbers of peer-reviewed publications and increased Psychological Capital, compared to the control group.

**Methods:**

The study included a 10-month intervention period and follow-up assessments occurring one, two, and three years after the intervention began. The intervention arm received a 10-month intervention with monthly meetings, near-peer mentoring, networking opportunities, and grant- and scientific-writing coursework. Participants in the control arm experienced the usual forms of mentoring, networking, and coursework that their institutions provided.

**Results:**

Of the 220 participants who completed the pre-intervention assessment (98% of all enrolled participants), 71% completed the post-intervention assessment at year 1, 60% at year 2, and 66% at year 3. Individuals in the intervention arm had significantly higher levels of self-efficacy, resilience, and optimism in the three years following the start of the intervention, compared to the control arm.

**Discussion:**

This finding suggests that the Building Up intervention can increase participants’ Psychological Capital.

## Introduction

The biomedical research workforce lacks diversity. In 2018, just 3.6% of medical school faculty members were Black or African American, and 3.2% were Hispanic or Latino, 19.2% were Asian, and 63.9% were White [2]. Approximately 35% of the Science, Technology, Education, or Medicine (STEM) workforce in 2018 were women [3], and the share of women decreases when moving upwards in rank or into leadership positions. Although approximately half of medical school students are women [4], only 28% of full professors in academic medicine, and 17% of medical school deans are women [5]. This lack of diversity negatively impacts scientific productivity. Research indicates that more diverse research teams can increase trust among patient populations and thereby improve recruitment [6]. Moreover, diverse teams publish in higher-impact journals and are cited more frequently [7,8]. Research studying gender-diverse scientific research teams has also indicated that having a gender-balanced group improves team performance [9].

Enrollment of underrepresented graduate students in STEM has increased in recent years, yet racial and ethnic disparities persist [3]. In 2021, 6.6% of doctoral students in science and engineering were Black or African American, 12.1% were Hispanic or Latino, 11.6% were Asian, and 64.8% were White [3]. Furthermore, it is well-documented that underrepresented individuals disproportionately leave the STEM research workforce [1,10]. Individuals from underrepresented backgrounds may encounter more deterrents as they attempt to progress through their research careers, compared to researchers from backgrounds that are not underrepresented [11]. Additionally, for early-career underrepresented faculty members, finding faculty role models and mentors with similar backgrounds is important but challenging at every career stage [12–17].

Given these challenges, underrepresented early-career researchers would benefit from “building up” their Psychological Capital. A Psychological Capital-building approach develops participants’ skill sets by focusing on four constructs: 1) *self-efficacy*, by building confidence through training in grant and manuscript writing; 2) *optimism*, by demonstrating success with near-peer mentors; 3) *hope*, by directing investigators towards their goals; and 4) *resilience*, by helping investigators bounce back from adversity [4]. Research suggests that improving early-career investigators’ Psychological Capital would lead to higher productivity and improved well-being [11]. This is based on research findings that Psychological Capital contributes to learning outcomes, increased engagement, and better coping behavior when faced with challenges [18]. Developing approaches to enhancing Psychological Capital has been shown to reduce the negative impact of systemic factors that disproportionately affect investigators [19] who are underrepresented in medicine. For example, Psychological Capital has been shown to buffer the negative impact of the well-documented “minority tax” whereby responsibilities for diversity and inclusion efforts are not shared by all but disproportionately burden residents [20] from groups that are underrepresented in medicine. However, mentoring and the Building Up of Psychological Capital has been shown to reduce the negative impact of this minority tax on scholars who are underrepresented in medicine.

The Building Up a Biomedical Research Workforce (Building Up) cluster-randomized controlled trial compares the effectiveness of an intervention to increase Psychological Capital and thereby increase research productivity among postdoctoral fellows and early-career faculty members. This is based on prior research showing that minoritized faculty face racism, isolation, diversity efforts disparities, clinical efforts disparities, lack of faculty development, and promotion disparities [21]. In fact, some argue that Psychological Capital is unequally distributed among people based on historical disparities across different social classes, ethnic backgrounds, race and gender [22]. It is important to address these disparities given the substantial research demonstrating the range of positive outcomes associated with the Building Up of Psychological Capital.

We hypothesize that participants from backgrounds underrepresented in science who are randomized to the intervention will have greater numbers of peer-reviewed publications, and they will have greater Psychological Capital than their colleagues randomized to the control group.

## Materials and methods

### Study setting and eligibility criteria

The trial, Building Up, was a multi-center, cluster-randomized, unblinded controlled trial at 25 academic medical institutions. These institutions range from large public institutions to smaller private institutions and are spread across the United States, from California to the East Coast, as well as northern and southern locations. The trial protocol was approved by a single Institutional Review Board at the University of Pittsburgh. Study recruitment has been previously described in detail [1]. Briefly, recruitment was done at the institution level and then at the participant level. Each participating institution had an assigned “site champion,” who was responsible for recruiting individuals.

To be eligible, participants (1) met the National Institutes of Health (NIH) definition for being underrepresented in biomedical sciences (i.e., individuals from racial or ethnic groups identified as underrepresented in biomedical sciences, individuals with disabilities, individuals from disadvantaged backgrounds, and women) [23]; (2) had a terminal degree (e.g., PhD, MD, PharmD); (3) were a postdoctoral fellow or an early-career faculty member in clinical and translational science; (4) intended to continue conducting clinical and translational research; and (5) had approximately 50% protected research time [1]. All participants applied to participate in Building Up and completed electronic informed consent.

### Study interventions

Building Up had one intervention arm and one control arm. Both arms received access to a monthly Excellence in Leadership webinar series designed to keep participants engaged. The intervention arm received a 10-month intervention consisting of four components: monthly meetings, mentoring, networking, and coursework. Participants in the intervention arm had monthly meetings with study-assigned near-peer mentors and fellow participants at their institution to discuss the academia’s hidden curriculum. They also received near-peer mentoring, networking opportunities, and grant- and scientific-writing coursework. Participants in the control arm experienced usual mentoring, networking, and coursework at their institution; these participants were expected to seek such opportunities on their own, as they were not provided by the study.

Sites were randomly assigned to the intervention or control arm using a fixed block size of two, to ensure equal allocation between the two arms. The study statistician performed randomization using SAS version 9.4 (SAS Institute, Cary, NC, USA) and informed the investigative team and study sites of randomization after recruiting the participants. No stratification was performed.

### Study outcomes

Participants completed online surveys and included CVs pre-intervention (Fall 2020) and again one year, two years, and three years later. The primary outcome, number of peer-reviewed original research publications in the three years following the start of the intervention, was extracted from CVs. For participants who did not submit CVs, a researcher used identifying information (i.e., name, institution, email address, and ORCID ID) collected during the study to search Google Scholar and ORCID for manuscripts published since the start of the intervention. We only reported peer-reviewed original research articles and systematic reviews and excluded case reports, editorials, commentary, and narrative literature reviews. We assessed the number of peer-reviewed original research publications with any author position, as first author, and as senior author.

Secondary outcomes include submitting an NIH grant proposal as a principal investigator (PI) and Psychological Capital. Participants indicated at the pre-intervention and annual follow-up assessments whether they had submitted a proposal in the past year for an NIH-funded grant as PI.

Participants also completed the validated, 24-item Psychological Capital Questionnaire at all survey timepoints [24]. Each of the four components of Psychological Capital (hope, self-efficacy, resilience, and optimism) was measured through six Likert scale questions. Response options ranged from 1 (strongly disagree) to 6 (strongly agree). Scores for questions within each component were summed, with a higher score indicating higher Psychological Capital.

### Statistical analysis

To compare the primary outcome (number of peer-reviewed publications with any author position during the three years following the start of the intervention) between the two arms, we used a negative binomial mixed model. Fixed effects included in the model were intervention arm, career stage, number of pre-intervention peer-reviewed publications, and highest degree. Random effects included institution, to adjust for possible clustering of scholars, and subject, to account for correlation among repeated measures for a participant. Highest degree was included in the model because it significantly differed by intervention arm (Table 1). We tested for an interaction between the intervention arm and time since the start of the intervention. We repeated this analysis with number of peer-reviewed publications as first author and senior author during the three years post-intervention as the outcomes, respectively.

**Table 1.** Pre-intervention characteristics of the Building Up a Biomedical Research Workforce Trial

	*n* (%)^a^		
Characteristic	Intervention (*n* = 108)	Control (*n* = 112)	*P-value*
Age, median (25^th–^75^th^ percentile)	35 (33–40)	37 (33–40)	0.46
Gender identity			0.20
Identifies as male	25 (23.2)	18 (16.2)	
Identifies as female^b^	83 (76.9)	93 (83.8)	
Race/ethnicity			0.10
Hispanic/Latinx	34 (31.5)	41 (36.6)	
Non-Hispanic/Latinx			
White	13 (12.0)	16 (14.3)	
Black	44 (40.7)	29 (25.9)	
Asian	8 (7.4)	18 (16.1)	
North African, Middle Eastern, or Multi-Racial	9 (8.3)	8 (7.1)	
Disability			0.40
Yes	4 (4.1)	7 (6.8)	
No	94 (95.9)	96 (93.2)	
Parent completed a bachelor’s degree			0.15
Yes	72 (66.7)	64 (57.1)	
No	36 (33.3)	48 (42.9)	
Years since highest degree achieved, median (25^th–^75^th^ percentile)	5 (3–11)	6 (3–8.5)	0.62
Career stage			0.39
Postdoctoral fellow	53 (49.1)	49 (43.8)	
Early career faculty	55 (50.9)	63 (56.3)	
Highest degree			0.03
MD	36 (33.3)	32 (28.6)	
PhD	56 (51.9)	74 (66.1)	
Other^c^	16 (14.8)	6 (5.4)	
Peer-reviewed publications, mean (standard deviation)			
All	8.9 (9.3)	10.0 (10.6)	0.37
First author	3.5 (4.2)	3.6 (3.7)	0.70
Senior author	0.4 (1.9)	0.5 (1.2)	0.20
Submitted NIH proposal as principal investigator	27 (24.8)	36 (31.3)	0.28
Psychological Capital, mean (standard deviation)			
Hope	26.4 (4.4)	25.9 (3.9)	0.21
Efficacy	26.4 (4.8)	26.5 (3.8)	0.80
Resilience	26.8 (3.9)	27.1 (3.9)	0.38
Optimism	26.0 (4.7)	25.2 (4.2)	0.07

NIH, National Institutions of Health.

a Unless otherwise specified.

b Includes participants who identify as transgender female.

c Includes DDS, DMD, DPT, DVM, EdD, MD/PhD, or PharmD.

The association between intervention arm and whether the participants submitted a proposal for an NIH-funded grant as PI was assessed using Poisson mixed models with robust error variance, controlling for intervention arm, career stage, highest degree, and pre-intervention submission of proposal for an NIH-funded grant as PI as fixed effects and institution and subject as random effects. Linear mixed models were used to compare the four Psychological Capital component scores between the two arms. Each model controlled for intervention arm, career stage, pre-intervention Psychological Capital component score, and highest degree as fixed effects and institution and subject as random effects. To confirm our assumption of minimal clustering effects in the power analysis, we calculated ICC for each outcome.

To assess robustness of our findings, we imputed missing data using multiple imputation with Markov Chain Monte Carlo methods, which assume that all variables in the imputation model have a joint multivariate normal distribution. Imputation models included all variables that were included in the original models, as well as age and race and ethnicity, which were associated with missing the outcome variables (an approach preferred versus the commonly used listwise deletion, which may introduce additional bias) [25].

We used SAS version 9.4 (SAS Institute, Cary, NC, USA) for all analyses. Reported p-values are two-tailed; p-values <0.05 were deemed statistically significant. Additional details can be found in the Supplementary Appendix.

## Results

### Response rate

**Figure 1.**
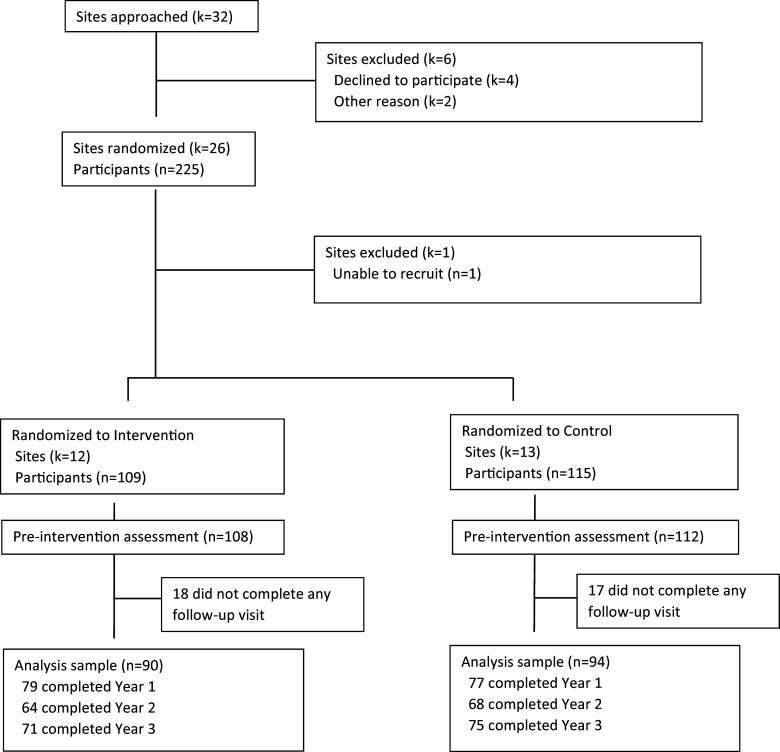
Institution and Participant Flow Diagram for the Building Up a Biomedical Research Workforce Trial.

Two hundred and twenty out of 224 (98%) enrolled participants completed the pre-intervention assessment. Of these, 84% (*n* = 184) completed at least one follow-up assessment at years 1–3 (83% of intervention and 82% of control arm) (Figure 1). Of participants who completed the pre-intervention assessment, 71% (*n* = 156) completed the post-intervention assessment at year 1, 60% (*n* = 132) at year 2, and 66% (*n* = 146) at year 3.

### Demographic measures

Seventy-nine percent of the cohort identified as female, 33% identified as Hispanic/Latinx, and 33% identified as Non-Hispanic Black. The type of highest degree achieved differed significantly by intervention assignment; a lower proportion of participants in the intervention arm had a PhD (52%) than in the control arm (66%; Table 1). No other demographic characteristics differed significantly by intervention arm.

### Association of intervention with primary and secondary outcomes

There was no statistically significant difference between the arms in number of peer-reviewed publications, or in the likelihood of submitting an NIH proposal as PI between the intervention and control groups, in the three years following baseline (Table 2). Individuals in the intervention arm had a significantly higher level of hope than those in the control arm one year following the start of the intervention [Beta (95% CI): 1.61 (0.41, 2.81); *p* = 0.01], but there was no significant difference between arms at two years [Beta (95% CI): 0.37 (−0.99, 1.72); *p* = 0.59] and three years [Beta (95% CI): −0.12 (−1.51, 1.26); *p* = 0.86] (Table 2). Individuals in the intervention arm also had a significantly higher level of self-efficacy [Beta (95% CI): 1.23 (0.38, 2.07); *p* = 0.005], resilience [Beta (95% CI): 0.95 (0.10, 1.79); *p* = 0.03], and optimism [Beta (95% CI): 0.96 (0.02, 1.90); *p* = 0.04)], compared to the control arm (Table 2). We confirmed our assumption of minimal clustering [ICC values for all outcomes were <0.01]. Results were attenuated with multiple imputation but remained statistically significant for self-efficacy and optimism (Supplementary Table 1).

**Table 2.** Association of Building Up intervention with primary and secondary outcomes

Outcomes	IRR	(95% CI)	*P-value*
Peer-reviewed publications			
All	1.15	(0.92–1.44)	0.23
First author	1.20	(0.89–1.63)	0.24
Senior author	0.68	(0.39–1.20)	0.19

	RR	(95% CI)	*P-value*
Submitted NIH proposal as PI	0.91	(0.67, 1.25)	0.56

	Beta	(95% CI)	*P-value*
Psychological Capital			
Hope			
Year 1	1.61	(0.41, 2.81)	0.01
Year 2	0.37	(−0.99, 1.72)	0.59
Year 3	−0.12	(−1.51, 1.26)	0.86
Efficacy	1.23	(0.38, 2.07)	0.005
Resilience	0.95	(0.10, 1.79)	0.03
Optimism	0.96	(0.02, 1.90)	0.04

CI, confidence interval; IRR, incidence rate ratio; NIH, National Institutions of Health; PI, principal investigator; RR, relative risk.

All models included person- and institution-level random intercepts and control for highest degree as a fixed effect..

### Primary and secondary outcomes over time

**Figure 2.**
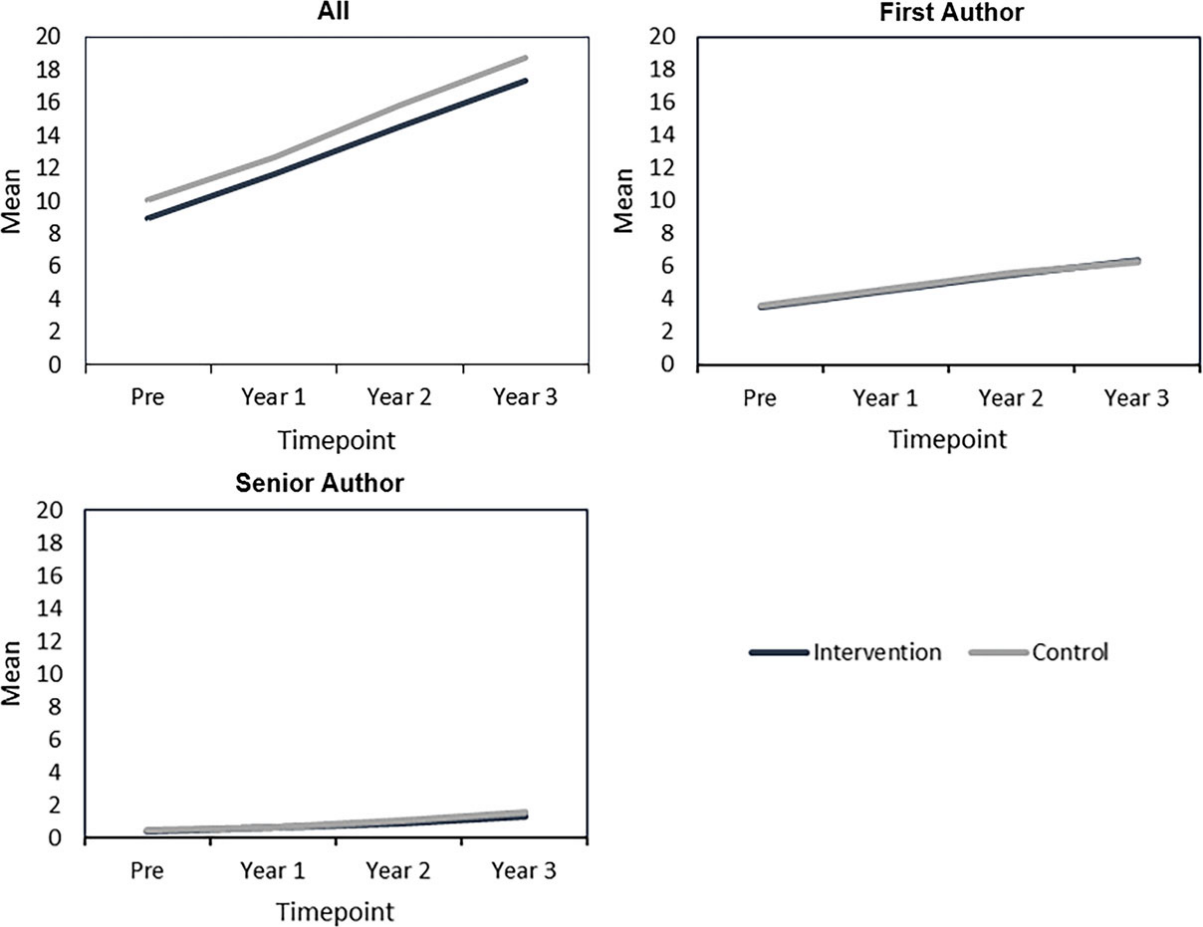
Number of peer-reviewed publications over time in Building Up intervention and control arms. Mean number of peer-reviewed publications at each timepoint is in Supplementary Table 2. P-values for comparisons of the number of peer-reviewed publications between different timepoints (year 1 versus pre-intervention and years 1-3) within the intervention arm and control arm, respectively, are in Supplementary Table 3. P-values are from linear mixed models with person- and institution-level random intercepts and control for highest degree as a fixed effect.

**Figure 3.**
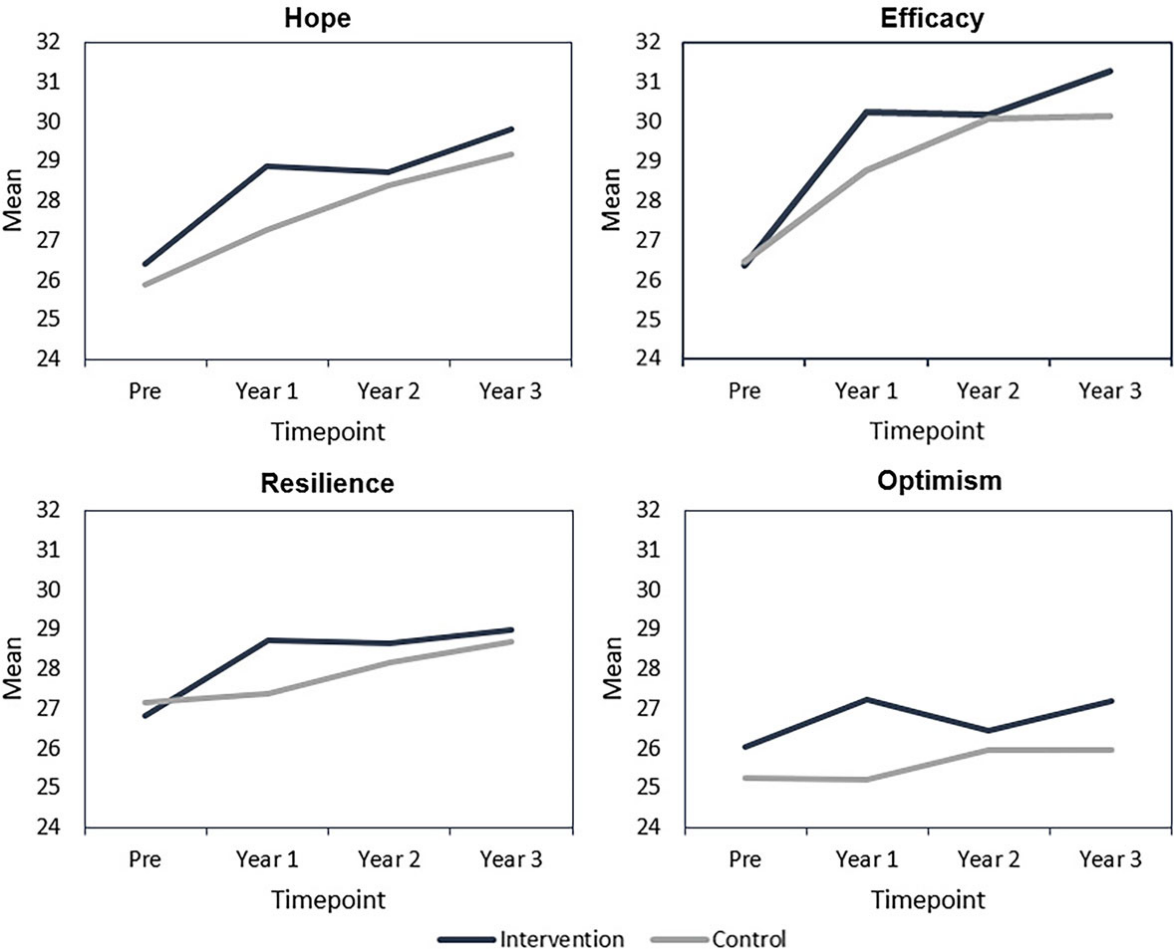
Psychological Capital over time in Building Up intervention and control arms. Mean Psychological Capital score at each timepoint is in Supplementary Table 2. P-values for comparisons of Psychological Capital components between different timepoints (year 1 versus pre and years 1-3) within the intervention arm and control arm, respectively are in Supplementary Table 3. P-values are from linear mixed models with person- and institution-level random intercepts and control for highest degree as a fixed effect.

The mean number of publications and scores for hope, self-efficacy, resilience, and optimism significantly increased pre-intervention to one-year in the intervention arm (Figures 2 and 3; Supplementary Tables 2 and 3). Only the mean number of publications and scores for hope and self-efficacy significantly increased at one-year in the control arm (Figures 2 and 3; Supplementary Tables 2 and 3). Mean number of publications and Psychological Capital component scores at each timepoint are in Supplementary Table 2. P-values comparing mean number of publications and Psychological Capital component scores at different timepoints within the intervention and control arms, respectively, are in Supplementary Table 3.

## Discussion

Our 25-site cluster-randomized trial of a 10-month career development intervention for underrepresented postdoctoral fellows and early-career faculty in academic research was successfully implemented, despite the onset of the COVID-19 pandemic [1]. We did not find a significant difference between the intervention and control groups in the number of peer-reviewed publications or grant applications during the three-year follow-up period. However, we found that the intervention group had significantly higher Psychological Capital in the areas of self-efficacy, resilience, and optimism, compared to the control arm.

The lack of significant publishing differences and grant submissions may be partly explained by the pandemic, which created research challenges and decreased productivity [26]. For example, women shouldered childcare responsibilities, disproportionately leaving the workforce and publishing less [27–30]. Lab-based researchers were unable to work in labs, which hindered their ability to conduct research, generate preliminary data for grant submissions, and publish. We based power calculations on pre-pandemic numbers of peer-reviewed publications per year. Due to the pandemic’s unforeseen consequences, the number of peer-reviewed publications was lower than anticipated, particularly among underrepresented researchers. This trend likely lowered the mean number of publications and the difference between groups, making it difficult to detect a difference between intervention arms.

We found that three Psychological Capital components – self-efficacy, resilience, and optimism – were significantly higher in the Building Up intervention arm compared to the control arm over the follow-up period. During the challenging COVID-19 pandemic, our Building Up program provided connection and support, as participants in the intervention met monthly with their peers [31].

Participants in the program also had opportunities to discuss another stressor – the murder of George Floyd and the subsequent protests. This context created a “dual stress” situation, with participants dealing with the pandemic alongside national social unrest [32]. This context may have influenced their Psychological Capital, adding another layer of stress that the intervention helped to address.

The intervention was originally intended to be delivered in person, and the shift to virtual delivery due to the pandemic may have reduced its potential impact. Nevertheless, we found a significant increase in Psychological Capital, which may reflect that mentoring and social support can buffer the effects of multiple stressors in academic medicine [33,34].

We found sustained improvement in self-efficacy, resilience, and optimism. A recent meta-analysis described sustained improvement in optimism and hope, but not self-efficacy and resilience [35]. We may have found sustained improvement in self-efficacy and resilience because of the sense of connectedness that was fostered during the monthly meetings. The intervention may have increased collective efficacy – the belief that the group can accomplish a task. This sense of collective efficacy may have interrupted negative thought patterns associated with stress, fostering a “we can do it” attitude [36].

Hope was also significantly higher in the intervention arm than in the control arm after the first year of the intervention, but this effect was not sustained in years 2 and 3. The initial boost in hope may have been related to the connection that participants felt with one another, generating a sense of agency (motivation). Additionally, the belief that the group could achieve its goals (a sense of agency) may have contributed to this sense of hope. Our findings align with previous research showing small to medium effects for Psychological Capital, based on effective interventions focused on well-being and performance outcomes [37].

Optimism and hope may be linked to long-term commitment to an institution or field. Role models, such as Building Up’s monthly speakers, could feed into optimism by showing participants that it is possible to succeed. Collective efficacy may also play a key role; when participants see others who share similar experiences, it strengthens their belief that they can achieve success, which can be seen as coping-specific self-efficacy that has a strong impact compared with general self-efficacy [38].

Importantly, there was no evidence of withdrawal or “quiet quitting” among participants, as measured by the fact that no participants withdrew from the study in either arm; this is an important trend when considering resilience. This lack of withdrawal suggests that the intervention helped foster sustained engagement, despite highly stressful conditions. This lack of quiet quitting, coupled with changes in Psychological Capital, evokes the notion of “quiet thriving,” or experiencing a sense of positive energy and personal growth, which can enhance personal investment and engagement.

This manuscript is subject to the following limitations. First, this study did not aim to compare the primary or secondary outcomes between participants from different backgrounds that are underrepresented in science (e.g., by racial or ethnic background or by gender) and was thus not powered to make such comparisons.

In the context of intensifying political resistance to diversity, equity, and inclusion (DEI) initiatives – including actions that have limited or prohibited such efforts in higher education and health systems – it is important to acknowledge concerns about the feasibility of implementing programs like Building Up. The current climate does present real and significant challenges: institutional leaders may fear repercussions, loss of funding, or political backlash for engaging in DEI-focused programming. Nevertheless, our experience suggests that it is still possible to carry out meaningful interventions by adapting them in ways that respond to specific contexts and still emphasize universal benefit. While Building Up was designed to address inequities faced by underrepresented groups, its structure – centered on evidence-based mentorship, skill-building, and professional development – has broad utility and applicability across faculty populations. Institutions may consider embedding such initiatives within broader faculty development or wellness efforts, thereby sustaining their integrity while remaining responsive to external constraints.

Moreover, programs like Building Up are not exclusive or exclusionary. They foster a research culture that supports all early-career investigators by strengthening Psychological Capital and professional networks. These outcomes are not only individually empowering but also institutionally advantageous. A more resilient and optimistic research workforce enhances scientific discovery, bolsters productivity, and contributes to financial sustainability through increased extramural funding and faculty retention [39–42]. The long-term benefits – including improved health equity, community engagement, and scientific innovation – are aligned with the mission of academic medicine, regardless of shifting political tides. As such, we argue that investing in this kind of career development programming remains both feasible and essential, even in an uncertain policy environment.

## Supporting information

10.1017/cts.2025.10144.sm001 Rubio et al. supplementary material Rubio et al. supplementary material

## Supplementary material

The supplementary material for this article can be found at https://doi.org/10.1017/cts.2025.10144.
