# Supplementary material for: Building Up a Biomedical Research Workforce Trial
Source: J Clin Transl Sci. 2025 Sep 10;9(1):e216. doi: 10.1017/cts.2025.10144 (PMC12529635; doi:10.1017/cts.2025.10144)
Supplement: Rubio et al. supplementary material [file S2059866125101441sup001.docx]

**Supplemental Table 1.** Association of Building Up intervention with primary and secondary outcomes after multiple imputation

| Outcomes | IRR (95% CI) | P |
| --- | --- | --- |
| Peer-reviewed publications |  |  |
| All | 1.17 (0.94-1.44) | 0.15 |
| First author | 1.09 (0.79-1.51) | 0.61 |
| Senior author | 0.87 (0.57-1.32) | 0.51 |
|  |  |  |
|  | Beta (95% CI) | P |
| Psychological Capital |  |  |
| Hope | 0.93 (-0.05, 1.92) | 0.06 |
| Efficacy | 1.03 (0.21, 1.85) | 0.03 |
| Resilience | 0.65 (-0.12, 1.41) | 0.10 |
| Optimism | 0.96 (0.07, 1.84) | 0.03 |
|  |  |  |
|  | RR (95% CI) | P |
| Submitted NIH proposal as PI | 0.92 (0.73, 1.16) | 0.47 |

CI, confidence interval; IRR, incidence rate ratio; NIH, National Institutes of Health; PI, principal investigator; RR, relative risk.

**Supplemental Table 2.** Observed Building Up a Biomedical Research Workforce Trial primary and secondary outcomes over time

| Outcomes | Pre-intervention | Year 1 | Year 2 | Year 3 |
| --- | --- | --- | --- | --- |
| **Intervention Arm** |  |  |  |  |
| Peer-reviewed publications, mean (std) |  |  |  |  |
| All | 8.9 (9.3) | 11.7 (10.8) | 14.5 (12.0) | 17.3 (13.4) |
| First author | 3.5 (4.2) | 4.5 (4.7) | 5.5 (4.9) | 6.4 (5.2) |
| Senior author | 0.4 (1.9) | 0.6 (2.5) | 0.9 (3.1) | 1.3 (3.7) |
| Psychological Capital, median mean (std) |  |  |  |  |
| Hope | 26.4 (4.4) | 28.9 (4.0) | 28.7 (4.1) | 29.8 (4.4) |
| Efficacy | 26.4 (4.8) | 30.2 (3.6) | 30.2 (3.3) | 31.3 (2.9) |
| Resilience | 26.8 (3.9) | 28.7 (3.2) | 28.7 (3.6) | 29.0 (3.5) |
| Optimism | 26.0 (4.7) | 27.2 (4.3) | 26.4 (4.3) | 27.2 (4.0) |
| Submitted NIH proposal as principal investigator, n (%) | 27 (24.8) | 26 (32.9) | 21 (32.8) | 20 (28.2) |
|  |  |  |  |  |
| **Control Arm** |  |  |  |  |
| Peer-reviewed publications, mean (std) |  |  |  |  |
| All | 10.0 (10.6) | 12.7 (11.9) | 15.9 (14.0) | 18.7 (15.9) |
| First author | 3.6 (3.7) | 4.6 (4.0) | 5.6 (4.8) | 6.3 (5.2) |
| Senior author | 0.5 (1.2) | 0.7 (1.6) | 1.1 (2.1) | 1.6 (2.8) |
| Psychological Capital, mean (std) |  |  |  |  |
| Hope | 25.9 (3.9) | 27.3 (4.4) | 28.4 (4.3) | 29.2 (4.2) |
| Efficacy | 26.5 (3.8) | 28.8 (3.3) | 30.1 (4.0) | 30.1 (4.3) |
| Resilience | 27.1 (3.9) | 27.4 (3.4) | 28.2 (3.4) | 28.7 (3.6) |
| Optimism | 25.2 (4.2) | 25.2 (4.0) | 26.0 (3.9) | 26.0 (4.1) |
| Submitted NIH proposal as principal investigator, n (%) | 36 (31.3) | 27 (35.1) | 28 (41.2) | 29 (38.7) |

NA, not applicable; NIH, National Institutions of Health; std, standard deviation

P-values comparing primary and secondary outcomes between different timepoints (year 1 versus pre and years 1-3) within the intervention arm and control arm, respectively are in Supplemental Table 3. P-values are from linear mixed models with person- and institution-level random intercepts and control for highest degree as a fixed effect.

**Supplemental Table 3.** Modeled^a^ Building Up a Diverse Workforce for Biomedical Research Trial primary and secondary outcomes over time

|  |  |  |  |  | P-value |  |
| --- | --- | --- | --- | --- | --- | --- |
|  | Pre-intervention | Year 1 | Year 2 | Year 3 | Year 1 vs.  Pre-intervention | Years 1-3 |
| **Intervention Arm** |  |  |  |  |  |  |
| Peer-reviewed publications, mean (95% CI) |  |  |  |  |  |  |
| All | 9.9 (7.7-12.1) | 12.3 (9.8-14.8) | 14.8 (12.1-17.5) | 16.9 (14.0-19.9) | <.001 | <.001 |
| First author | 3.7 (2.7-4.8) | 4.6 (3.5-5.6) | 5.5 (4.4-6.5) | 6.2 (5.0-7.4) | <.001 | <.001 |
| Senior author | 0.6 (0.2-1.0) | 0.8 (0.3-1.3) | 1.0 (0.4-1.6) | 1.3 (0.6-2.0) | 0.01 | <.001 |
| Psychological Capital, mean (95% CI) |  |  |  |  |  |  |
| Hope | 26.4(25.3-27.4) | 28.9(27.8-29.9) | 28.9(27.8-30.0) | 29.4(28.2-30.7) | <.001 | 0.36 |
| Efficacy | 26.1(25.1-27.2) | 29.9(29.0-30.9) | 29.9(28.9-30.9) | 30.9(30.0-31.7) | <.001 | 0.004 |
| Resilience | 26.6(25.6-27.5) | 28.3(27.4-29.2) | 28.6(27.6-29.5) | 28.6(27.6-29.6) | <.001 | 0.59 |
| Optimism | 26.4(25.2-27.5) | 27.2(26.1-28.4) | 27.0(25.7-28.2) | 27.3(26.1-28.4) | 0.03 | 0.71 |
| Submitted NIH proposal as PI, % (95% CI) | 20.1(12.8-27.4) | 24.7(15.5-33.8) | 24.7(14.8-34.7) | 21.2(12.4-29.9) | 0.29 | 0.47 |
|  |  |  |  |  |  |  |
| **Control Arm** |  |  |  |  |  |  |
| Peer-reviewed publications, mean (95% CI) |  |  |  |  |  |  |
| All | 14.4 (11.7-17.2) | 16.7 (13.8-19.6) | 19.5 (16.4-22.7) | 22.1 (18.7-25.5) | <.001 | <.001 |
| First author | 4.3 (3.2-5.3) | 5.0 (3.9-6.1) | 5.9 (4.7-7.1) | 6.5 (5.2-7.8) | <.001 | <.001 |
| Senior author | 1.2 (0.9-1.5) | 1.4 (1.1-1.8) | 1.8 (1.4-2.2) | 2.3 (1.7-2.8) | <.001 | <.001 |
| Psychological Capital, mean (95% CI) |  |  |  |  |  |  |
| Hope | 25.6(24.3-26.8) | 26.9(25.5-28.3) | 28.0(26.6-29.4) | 29.1(27.7-30.5) | 0.02 | <.001 |
| Efficacy | 25.3(24.2-26.4) | 27.6(26.5-28.8) | 28.7(27.4-30.0) | 28.9(27.6-30.2) | <.001 | 0.01 |
| Resilience | 26.6(25.4-27.8) | 26.9(25.6-28.1) | 27.6(26.4-28.8) | 28.2(27.0-29.4) | 0.51 | 0.01 |
| Optimism | 25.1(23.7-26.4) | 25.1(23.7-26.4) | 25.9(24.5-27.2) | 26.0(24.6-27.5) | 0.99 | 0.04 |
| Submitted NIH proposal as PI, % (95% CI) | 34.3(21.6-47.0) | 37.9(24.6-51.2) | 45.0(29.4-60.6) | 40.9(27.3-54.4) | 0.56 | 0.65 |

CI, confidence intervals; NIH, National Institutions of Health; PI, principal investigator

^a^ P-values compare primary and secondary outcomes between different timepoints (year 1 versus pre and years 1-3) within the intervention arm and control arm. We used linear mixed models with person- and institution-level random intercepts and control for highest degree as a fixed effect to estimate means (95% CI) at each timepoint.
